# Psychometric Properties of the CEVEO Bullying Subscales for Aggressors in School and Leisure Contexts Among Chilean Adolescents: Profiles Based on Moral Disengagement, Aggression Frequency, and Context

**DOI:** 10.3390/children13010134

**Published:** 2026-01-16

**Authors:** Karina Oñate-Hormazábal, Beatriz Pérez, Andrés Concha-Salgado

**Affiliations:** 1 Departamento de Psicología, Universidad de La Frontera, Temuco 4780000, Chile; k.onate03@ufromail.cl (K.O.-H.); andres.concha@ufrontera.cl (A.C.-S.); 2 Facultad de Psicología, Universidad de Oviedo, Plaza Feijoó S/N, 33003 Oviedo, Spain

**Keywords:** bullying, psychometric properties, adolescents, moral disengagement, perpetrator profiles

## Abstract

**Highlights:**

**What are the main findings?**

• The aggression subscales of the CEVEO demonstrate adequate psychometric properties for use with Chilean adolescents, enabling the assessment of aggression in both school and leisure contexts.
• These subscales allow for the classification of aggressors into distinct types based on the frequency and context of their aggressive behaviors.

**What are the implications of the main findings?**

• A high-risk profile (Profile 3) was identified, consisting of adolescents who exhibited a high frequency of aggression across both contexts (school and leisure) and elevated levels of moral disengagement.
• Measuring aggression in both school and leisure settings allows for a more comprehensive identification of high-risk aggressor profiles, as those involved in multiple contexts show higher levels of moral disengagement regardless of frequency.

**Abstract:**

**Background:** Adolescent violence occurs both within and beyond the school setting. Furthermore, risk factors for aggression, such as Moral Disengagement (MD), do not operate uniformly and may be triggered in one context but not another. This highlights the need for instruments that assess aggression’s manifestation across contexts to enable a more comprehensive understanding of the phenomenon. **Objective:** To assess the psychometric properties of the Bullying at School and Bullying during Leisure subscales from the Questionnaire for Assessing Peer Violence in School and Leisure Settings (CEVEO) in Chilean adolescents, and to examine differences in MD among perpetrator profiles based on both frequency and context of aggression. **Method:** Instrumental, multivariate, cross-sectional, quantitative, and correlational design. The sample comprised 864 Chilean students (M age = 15.4; SD age = 1.3). Girls represented 58% of the sample. **Results:** A 13-item unifactorial model was supported for both subscales, with good internal consistency. Scores correlated positively with MD, and boys scored higher than girls on both subscales. Three profiles were identified: (1) no high aggression; (2) high aggression in one context; and (3) high aggression in two contexts. MD increased with the number of contexts, regardless of aggression frequency. **Conclusions:** Findings provide validity evidence for the CEVEO bullying subscales in Chilean adolescents, based on their internal structure, associations with external variables, and reliability. The instrument is useful for detecting violence across settings and identifying profiles based on the contextual extent of aggression.

## 1. Introduction

School bullying is a deliberate and repetitive aggressive behavior meant to cause another person harm, typically due to a power imbalance [1,2,3]. This phenomenon is a widespread problem, with boys tending to be more frequent perpetrators. Regarding victimization, findings are mixed and appear to depend on the type of bullying considered [4,5,6,7,8,9].

It is estimated that all students have been exposed to bullying at some point in their school experience as bystanders, bullies, and/or victims [10]; and although prevalence data differ among countries, it is estimated that almost one-third of the world’s adolescents have been victims of bullying [2,11,12]. In Chile, the national survey on bullying in schools [13] found that 61% of respondents had been victims of verbal abuse (insults, mockery, or threats), 41% had experienced episodes of social isolation, humiliation, and negative gossip about them, and 33% had been physically intimidated (hitting or breaking their things). This phenomenon is on the rise, as complaints about bullying after the COVID-19 pandemic increased by 59.4% nationwide [14].

### 1.1. The Role of Moral Disengagement in School Violence

The origins of school violence are multifactorial and often attributed to the interaction between the individual and their environment, with risk factors present at multiple levels. For example, factors such as structural violence, exposure to family violence, the quality of the student–teacher relationship, and individual traits like assertiveness and empathy [6,15]. Among them, recent scientific literature has focused on the link between bullying/cyberbullying and moral disengagement (MD) [4,16,17,18], a dynamic individual risk factor with potential for consideration in intervention programs.

MD is a process of cognitive restructuring that modifies the interpretation and evaluation of moral standards, favoring the justification of ethically reprehensible behaviors toward others without guilt or shame [19]. The literature suggests that aggressive behavior towards peers is associated with the activation of MD mechanisms as a strategy to distance oneself from moral transgressions and prevent self-censorship [16]. In fact, it is an anticipatory factor of different types of violence in children and adolescents described as a key predictor of maladaptive behaviors. Examples of this include violence in dating relationships [20], child–parent violence [21], and violent antisocial behavior [22].

Existing scientific evidence consistently supports the proposition that MD mechanisms are associated with bullying and cyberbullying [23,24]. However, to understand the relationship between these variables, it is important to keep in mind that MD does not operate automatically or uniformly, and its activation depends on internal and external factors [25,26]. For example, high levels of anger rumination favor the relationship between MD and bullying [27]; a negative family or neighborhood environment facilitates MD [28], whereas a positive school environment reduces it [29]. This highlights the need to differentiate bullying according to the context, as dynamics and motivations may differ between contexts.

### 1.2. Leisure-Time Aggression in Adolescents

School is an excellent meeting place for children and adolescents, a space for coexistence built around social norms and hierarchies that emanate both from the institution and the students. Hence, it is understandable that the school context becomes a setting of particular interest. However, although the school environment may be conducive to the development of school bullying, it can transcend its structure and control mechanisms [30].

The work by Díaz-Aguado et al. [10] reported that 41% of the violence young people perceive occurs in leisure settings, that is, outside the school, mainly on weekends. On the other hand, Ahn et al. [31] demonstrated through census data that the incidence of violence among adolescents is higher outside of school. The studies by Pulido [32] and Pulido et al. [33] indicate that students who exhibit aggressive behavior at school often do so during their leisure as well. Moreover, these studies establish distinct profiles of both victims and aggressors based on the severity of violence experienced in school and leisure contexts. A particularly high-risk group is identified, characterized by elevated scores in both settings and involvement in one or both roles [33].

Given that situational factors such as school climate and social support can moderate the use of MD mechanisms [29], it is possible that perpetrators who exhibit aggressive behavior across different contexts (school and leisure) are exposed to conditions that reinforce the perceived legitimacy of harm in each setting. The persistence of bullying beyond the school environment suggests a greater stability of MD as a mechanism for regulating aggressive behavior, potentially making these perpetrators less responsive to interventions that focus exclusively on the school context. Therefore, a differentiated approach that considers the specific contexts in which bullying occurs is essential for developing more effective preventive and therapeutic strategies.

### 1.3. Bullying Measurement Scales

Self-report scales are the most commonly used method for measuring bullying. Vivolo-Kantor et al. [12] and Xie et al. [34] located 41 and 75 different measures of bullying in the international scientific literature, respectively. The two research groups coincide in pointing out the inconsistency among instruments in terms of construct definitions, components, and the timeframes being assessed. Additionally, Herrera-López et al. [35] conducted a bibliometric study on bullying research in Latin America, identifying the lack of instruments specifically developed for, or validated in, Latin American contexts as a major limitation.

In Chile, we have four studies on the adaptation of scales and analysis of their psychometric properties (see Table 1): Internet Experiences Questionnaire (IEQ, Lecannelier et al., 2010), School Peer Bullying (Maltrato entre Iguales por Abuso de Poder para escolares [MIAP]) [36], Bullying and Victimization Scales (Escalas de Agresión y Victimización [EAV]) [5], and the aforementioned Olweus Bully/Victim Questionnaire (OBVQ-R) [1].

The study by Gaete et al. [1] stands out among the above studies who worked on the adaptation and study of the psychometric properties of the OBVQ-R. The BVQ [38] -and its derivatives- is considered one of the most widespread, influential, and internationally recommended instruments [1,5,10,34,36]. This scale assesses bullying-related events among peers at school and online in the past two months, based on Olweus’ [38] definition of bullying, as outlined at the beginning of this paper. It includes two subscales: victimization and bullying. The scale demonstrates evidence of reliability and validity regarding its internal structure, its relationship with other variables, and its concurrent validity for use with Chilean schoolchildren. Given the above, the OBVQ-R [1] emerges as a valid and reliable alternative for measuring school bullying in Chile. Moreover, the internationalization of the measure allows cross-cultural comparisons, for instance, with countries such as Argentina or Brazil. However, neither the OBVQ-R nor the other instruments reviewed allow for the assessment of adolescent violence during leisure time. This limitation highlights the need for a measurement tool with adequate psychometric properties for use with Chilean youth—one that can assess violence in both school and leisure contexts.

The Questionnaire for Assessing Peer Violence in School and Leisure Settings (Cuestionario de Evaluación de la Violencia entre Iguales en la Escuela y en el Ocio [CEVEO]) [10] measures the phenomenon of peer violence (PV) across both contexts, based on the premise that young people may engage in similar behaviors in school and leisure environments. A modified version of this instrument has demonstrated adequate psychometric properties for use with Spanish and Mexican youth populations [39], making it a potentially suitable alternative to address the measurement gaps in Chile.

### 1.4. Study Objectives

Considering the need for a scale in Chile to measure adolescent aggression both within and outside the school setting, the primary objective of the present study was to evaluate the psychometric properties of the bullying subscales of the CEVEO in a sample of adolescents in southern Chile. From this first objective, the following specific objectives are derived: (1) to provide evidence of validity based on internal structure; (2) to provide evidence of reliability; (3) to provide evidence of validity based on the relationship with other variables: moral disengagement and sex.

To identify perpetrator profiles with varying levels of complexity based on their moral disengagement scores, our second objective was to analyze differences in MD among perpetrator profiles, considering both the frequency of aggression and the number of contexts in which it occurs. From this objective, the following specific aims were derived: (4) to identify perpetrator profiles based on the high-frequency of aggression and the number of contexts in which it is perpetrated (none, one, or two); (5) to compare MD scores across perpetrator profiles; and (6) to determine whether MD scores differ according to the number of contexts in which aggression is perpetrated, after controlling for aggression frequency.

Based on the original instrument, we hypothesize that both subscales will exhibit a two-factor structure (H1a and H1b) and reliability coefficients above 0.70 (H2a and H2b). Additionally, we expect both bullying measures to correlate significantly and positively with moral disengagement, and that bullying levels will be higher among boys in both contexts (H3a and H3b). Regarding the second objective, we hypothesize that aggressor profiles will differ in the frequency and patterns of aggressive behavior, with higher levels expected among those who display such behavior in two contexts compared to those who do so in only one. (H4); that profiles characterized by higher aggression frequency and greater contextual spread will exhibit higher MD scores (H5); and that the number of contexts in which aggression is perpetrated will be positively associated with MD scores, independently of aggression frequency (H6).

## 2. Materials and Methods

### 2.1. Design

This work combines an instrumental study, focusing on the study of the psychometric properties of the CEVEO [40], and a multivariate quantitative correlational study.

### 2.2. Participants

The target population consisted of adolescents attending educational institutions in the La Araucanía and Biobío regions of southern Chile. A non-probability, convenience sampling method was used. The sample size was estimated using an online calculator [41], based on the following parameters: (a) a small effect size of 0.10, (b) a statistical power of 0.80, (c) two latent variables, (d) 28 observed variables (i.e., scale items), and (e) a significance level of 0.05. The recommended sample size was 947 adolescents. The final sample comprised 864 participants. Although the sample fell slightly short of the expected size, there is broad agreement that factor analysis requires a minimum of 500 participants [42].

As for the characteristics of the sample (see Table 2), 58% were girls, with an average age of 15.35 years (SD = 1.3). The 52.3% were in Araucanía and 47.7% in Biobío. According to the Ministerio de Desarrollo Social y Familia [43], both regions in southern Chile are above the national poverty average. Araucanía is the second poorest region in the country and the second region with the highest presence of people belonging to an indigenous people (15.3%). The Biobío Region is the ninth poorest region, and the fourth with the highest presence of people belonging to the Mapuche people (7%). Furthermore, at the national level, the poverty rate among individuals who identify as belonging to an Indigenous group (8.8%) was statistically significantly higher than that of the rest of the population (6.2%) in 2022 [44].

### 2.3. Instruments

#### 2.3.1. Ad Hoc Sociodemographic Questionnaire

This questionnaire recorded sociodemographic information on the participants, such as their age, gender, origin, ethnicity, socioeconomic level, and type of school.

#### 2.3.2. Bullying at School Subscale of the CEVEO

Bullying at School subscale of the Questionnaire for Assessing Peer Violence in School and Leisure Settings (CEVEO) [10], assesses violent behaviors occurring at school and during leisure over the past two months. The subscale comprises 15 items in a four-point Likert-type response format, from never (1) to very much (4), with “sometimes” as the minimum frequency category, thereby explicitly capturing the repetition of behaviors. This subscale is organized in a two-factor structure based on severity (exclusion and bullying of medium severity, and severe bullying at school). The higher the score, the greater the bullying at school. This questionnaire presents psychometric evidence for its use with Spanish and Mexican adolescents, with Cronbach’s alpha values between 0.86 and 0.93.

#### 2.3.3. Bullying During Leisure Subscale of the CEVEO

The Bullying During Leisure subscale of the CEVEO [10] consists of 13 items rated on a four-point Likert-type scale, ranging from 1 (never) to 4 (very much). The subscale has a two-factor structure based on severity, distinguishing between moderate bullying and social exclusion, and severe bullying during leisure. Higher scores indicate greater involvement in bullying in leisure contexts. This instrument explicitly considers the repetition of behaviors, assessing the frequency of violent acts occurring over the past two months, with “sometimes” as the minimum frequency category. The questionnaire has demonstrated strong psychometric properties when used with Spanish and Mexican adolescents, with Cronbach’s alpha values ranging from 0.86 to 0.93.

#### 2.3.4. Mechanisms of Moral Disengagement Scale

The MMDS was developed by Bandura et al. [45] to assess the eight theoretical mechanisms of moral disengagement (MD). In this study, we used the brief version adapted for Chilean adolescents [22]: a unidimensional self-report questionnaire consisting of 10 Likert-type items with five response options, ranging from strongly disagree (1) to strongly agree (5). Higher scores indicate higher levels of MD. In the adaptation study, McDonald’s omega was 0.862 for sample 1 and 0.865 for sample 2 [22]. In the present study, the internal consistency of the items was 0.844.

### 2.4. Procedure

First, the CEVEO underwent a process of cultural and linguistic adaptation by consulting 3 researchers with experience living in both socio-cultural settings. After the modification of certain expressions (e.g., replacing pegándole [hitting] with golpeándole [“beating”]) and the approval of the relevant educational authorities in each school, the complete questionnaire was tested in a pilot study (*n* = 56). No further changes were necessary.

For the final administration, the project researchers contacted the Municipal Education Departments of each district to request their collaboration in the recruitment process. These departments then reached out to schools, inviting them to participate, and schools that expressed interest contacted the research team. Subsequently, online meetings were held with each participating school to present the project, the data collection procedures, and compensation. Within the participating schools, potential participants were invited to take part during class time, where they received an information letter to share with their parents or guardians. Once parental/guardian consent and student assent were obtained, data collection was carried out in groups within the classroom setting, using paper-and-pencil surveys. The study was reviewed and approved by the Ethics Committee of Scientific of the University of La Frontera.

### 2.5. Plan of Analysis

For the descriptive approach to the items, the response percentage for each category, the mean, standard deviation, skewness, and kurtosis were calculated. The corrected item-total correlation was also evaluated, assuming acceptable values from 0.3 and above. To respond to specific objective 1, the fit of the structure of both scales was tested in the study sample using a confirmatory factor analysis (CFA). The WLSMV estimator was selected because it is specifically designed for categorical or ordered data, does not assume multivariate normality, provides robust standard errors, and yields more accurate parameter estimates and fit indices than ML-based estimators when item distributions are skewed or kurtotic [46]. The comparative fit index (CFI), the Tucker–Lewis index (TLI), and the root mean square error of approximation (RMSEA) were included as indices of fit. Model fit was considered good when CFI and TLI were ≥0.95 and RMSEA ≤ 0.05, and reasonable when CFI and TLI were ≥0.90 and RMSEA ≤ 0.08 [47,48].

In addition, the convergent validity of the items of each factor was analyzed by average variance extracted (AVE), the acceptance criterion of which is ≥0.50 since the construct must share more than half of the variance with its items. Likewise, construct reliability (CR) was determined, which has as its criterion CR ≥ 0.70, indicating that the construct theoretically represents what it intends to measure [49]. The discriminant validity (DV) was also calculated to determine if the estimates of the AVE for two factors are higher than the estimate of the squared correlation between those two factors. This indicates that a dimension explains the variance of its items more than it shares with another dimension [50].

Subsequently, to respond to Specific Objective 2, the reliability of the subscales was obtained using three variants of the alpha coefficient: standardized Cronbach’s alpha, McDonald’s omega, and ordinal alpha [51]. For the study of validity evidence based on the relationship with other variables that respond to Objective 3, the Pearson’s *r* correlation coefficient between the CEVEO subscales and the MMDS scale was used. For the discriminant validity analysis, Student’s *t*-test was used to compare the means between boys and girls in the scores obtained on both subscales of the CEVEO. In addition, the effect size was calculated using Cohen’s *d*.

To address Specific Objective 4, frequency analyses were performed, extreme groups were identified based on their position within the quartile distribution, and one-way ANOVAs with the Games–Howell post hoc test applied due to unequal variances between groups were conducted to compare the identified profiles in terms of aggression frequency at school and during leisure time. For Specific Objective 5, one-way ANOVAs were also conducted, followed by Games–Howell post hoc tests where appropriate, to examine differences between the previously identified groups. Finally, to address Specific Objective 6, an analysis of covariance (ANCOVA) was conducted, using the Games–Howell post hoc test and controlling for the effects of aggression frequency at school and during leisure. Effect sizes were calculated using Cohen’s d and η^2^ to interpret the magnitude of the observed effects.

## 3. Results

### 3.1. Descriptive Analysis of the Items

A tendency to report the response option “never” most frequently was noted, resulting in a positive skewness and a leptokurtic distribution in all items. The school bullying items with the lowest frequency of behavior were 14 and 12, with 98.7% and 98.4% of never responses, respectively. In contrast, the highest frequency reported corresponds to items 2 and 4, which obtained 8.8% and 8.6% of the responses, respectively, after combining the percentages of the responses often and very often (see Table 3).

Coincidentally, in the Bullying during Leisure subscale, the items that showed the lowest frequency of behavior were 11 and 12, with 98.6% and 98.5% of never responses, respectively. Similarly, the behaviors with the highest frequency were the same as those observed at school, with 6.8% for item 2 and 6.2% for item 4 after merging the categories often and a lot (see Table 3).

### 3.2. Bullying at School Subscale

**Figure 1:**
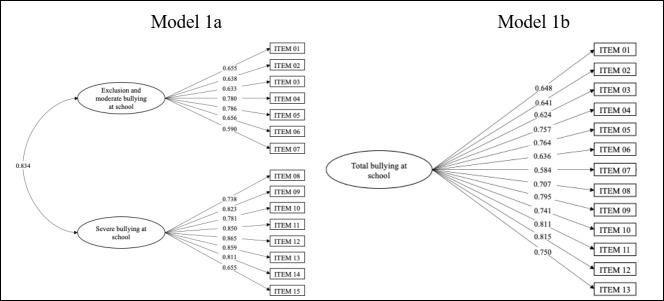
Models 1a (bifactorial) and 1b (unifactorial) of the bullying at school subscale.

For the subscale of Bullying at School, the results of model 1a showed acceptable values in the fit indices of the 2-factor model (CFI = 0.933, TLI = 0.921, RMSEA = 0.051, and SRMR = 0.081). The factor weights ranged from 0.633 to 0.786 for factor 1 and 0.655 to 0.865 for factor 2. The correlation between the two factors is greater than 0.8 (see Figure 1). The AVE for factor 1 was below the cut-off point of 0.50. For factor 2, on the other hand, the AVE was above 0.50. The CR for both factors was above 0.70, which represents an adequate reliability value. On the other hand, when studying the DV index, it was found that none of the AVE estimates was greater than the squared correlation between the two factors, meaning neither factor differed sufficiently from the other, affecting their DV (see Table 4).

In the one-factor model (M1b), two items were eliminated due to their extreme kurtosis (item 14 = 163.82, item 15 = 82.70) and corrected item-total correlation lower than 0.3 (see Table 3). With 13 items, the model presented acceptable values in the fit indices for model 1b (CFI = 0.932, TLI = 0.918, RMSEA = 0.058 and SRMR = 0.063). Factor loadings ranged from 0.584 to 0.815 (see Figure 1). The AVE was higher than 0.50, and the CR was 0.70, representing adequate values of convergent validity of the items (see Table 4).

### 3.3. Bullying During Leisure Subscale

**Figure 2:**
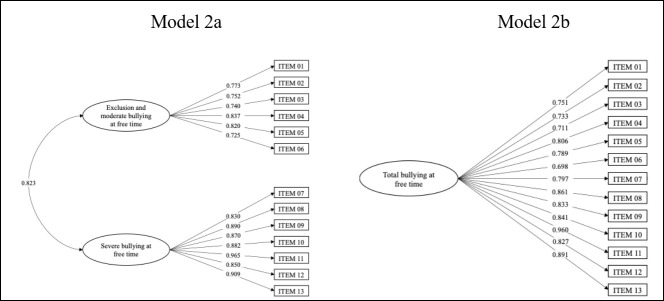
Models 2a (bifactorial) and 2b (unifactorial) of the Bullying during Leisure subscale.

For the Bullying during Leisure subscale, the results of the two-factor model (2a) showed reasonable values for the fit indices for the two-factor model (CFI = 0.965, TLI = 0.957, RMSEA = 0.056, and SRMR = 0.070). Factor weights ranged from 0.725 to 0.837 for factor 1 and from 0.850 to 0.965 for factor 2 (see Figure 2). The AVE were above 0.50 and the CR were above 0.70, both adequate. However, when studying the DV, it was again found that only one of the AVE estimates was greater than the squared correlation estimates of the two factors. Following the logic that a latent construct should explain more variance in the measures of its items than what it shares with another factor, discriminant validity could not be demonstrated for either dimension of the Bullying during Leisure subscale (see Table 4).

For the unifactorial model (2b), acceptable values were obtained for the fit indices (CFI = 0.948, TLI = 0.937, RMSEA = 0.068, and SRMR = 0.091). The factor weights ranged from 0.698 to 0.960 (see Figure 2). The AVE was above 0.50, and the CR was above 0.70, representing adequate values for the unifactorial model of bullying in leisure activities (see Table 4 and Figure 2). The results show that the bifactorial hypotheses (H1a and H1b) are not fulfilled, but they do support the unifactorial interpretation of the subscales.

The correlation between the two subscales was significant, positive, and strong (*p* < 0.001, *r* = 0.585), indicating that high levels of school aggression are associated with higher scores in the leisure context.

### 3.4. Reliability

With a standardized Cronbach’s alpha coefficient of 0.926, a McDonald’s omega of 0.926, and an ordinal alpha of 0.931, the Bullying at School subscale showed high internal consistency of the items. For the Bullying during Leisure subscale, a standardized Cronbach’s alpha coefficient of 0.951, a McDonald’s omega of 0.951, and an ordinal alpha of 0.961 were obtained. Both analyses confirm hypothesis 2.

### 3.5. Evidence of Validity Based on the Relationship with Other Variables

The subscales of Bullying at School (*p* < 0.001, *r* = 0.255) and Bullying during Leisure (*p* < 0.001, *r* = 0.233) showed significant, positive, small correlations with the total MD score. This made it possible to demonstrate hypothesis 3a, providing evidence of convergent validity of the bullying subscales of the CEVEO.

On the other hand, the means of bullying at school were 16.67 (SD = 3.83) in boys and 15.42 (SD = 3.36) in girls. In bullying during leisure, boys averaged 15.71 points (SD = 3.96) versus 14.66 (SD = 2.97) for girls. The differences were statistically significant and of small effect size at both schools (t = 5.050, *p* < 0.000, Cohen’s d = 0.348) and leisure time (t = 4.430, *p* < 0.001, Cohen’s d = 0.304). These results corroborate hypothesis 3b, as both subscales can discriminate between boys and girls regarding the reported frequency of manifesting aggressive behaviors, which is higher in boys.

### 3.6. Aggressor Profiles Based on Frequency and Contexts of Violence

A total of 20.6% (n = 178) of the participants reported not engaging in violence in any context. With at least a minimum frequency of aggression—sometimes, in the last two months-, 52.5% (n = 454) reported engaging in violence in both contexts and the remaining 26.9% (n = 232) reported aggression in only one of the two contexts.

In line with Specific Objective 4, groups of participants with the highest levels of aggression at school and during leisure time were identified. More specifically, aggression scores were divided into quartiles, and participants in the highest quartile (Quartile 4) were selected. Subsequently, aggressor profiles were constructed based on the contexts of action: (1) no high aggression; (2) high aggression in one context; and (3) high aggression in two contexts (see Table 5). Table 5 also presents the descriptive data for Group 2, divided into two subgroups: (2.1) High aggression in the school context and (2.2) High aggression in leisure time.

**Figure 3:**
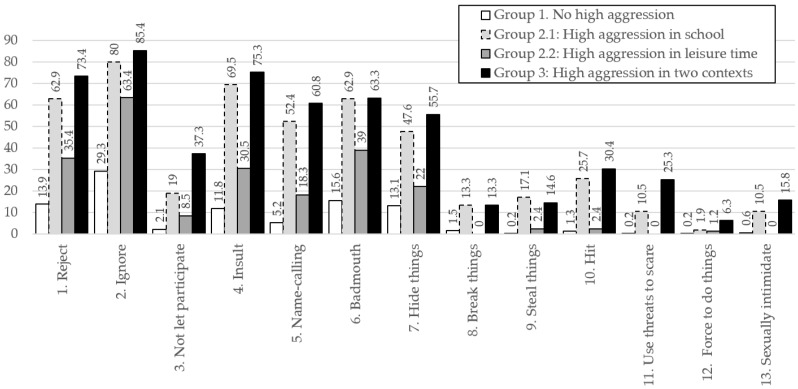
Percentage of participants per group who reported engaging at least “sometimes” in each aggressive behavior at school during the past two months.

**Figure 4:**
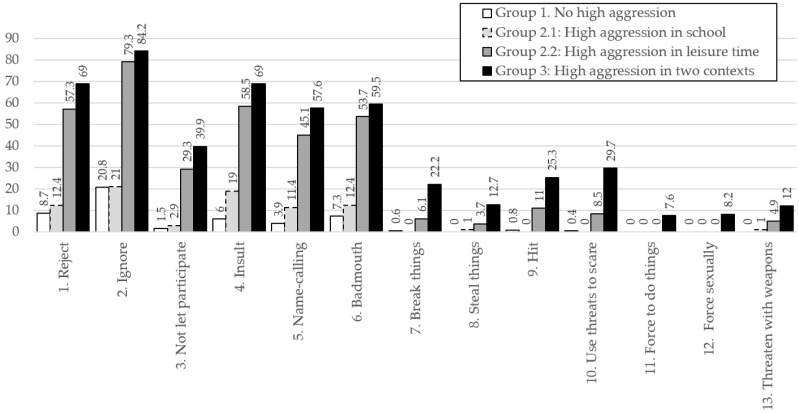
Percentage of participants per group who reported engaging at least “sometimes” in each aggressive behavior at leisure time during the past two months.

The profiles 1, 2.1, 2.2 and 3 differed significantly in aggression frequency at school, F(3, 860) = 402.95, *p* < 0.001, η^2^ = 0.58, and during leisure time, F(3, 860) = 355,12 *p* < 0.001, η^2^ = 0.55, indicating large effect sizes. Comparisons of school aggression levels between Groups 2.1 and 3 (t = −3.77, df = 260.98, *p* = 0.001, d = 0.44), and of leisure aggression levels between Groups 2.2 and 3 (t = −5.67, df = 232.89, *p* < 0.001, d = 0.66), revealed statistically significant differences in both cases, with small and medium effect sizes, respectively. Thus, individuals with high levels of aggression in both contexts showed higher aggression at school than those with elevated aggression limited to school, and higher aggression during leisure than those with elevated aggression limited to leisure. Figure 3 and Figure 4 present the response patterns of Groups 1, 2.1, 2.2, and 3. These results support H4.

### 3.7. Comparison of Aggressor Profiles in MD

A one-way ANOVA was conducted to compare levels of MD among the three groups of participants. The results revealed statistically significant differences between groups 1, 2, and 3, F(2, 861) = 42.91, *p* < 0.001, η^2^ = 0.091, indicating a medium effect size. Levene’s test indicated that the assumption of homogeneity of variances was violated, F(2, 861) = 4.377, *p* = 0.013. Therefore, post hoc comparisons using the Games–Howell test were conducted to correct for variance heterogeneity.

The results showed that group 1 scored significantly lower than group 2, t = −5.77, df = 329.47, *p* < 0.001, d = 0.492, and group 3, t = −7.721, df = 228.28, *p* < 0.001, d = 0.820. In addition, group 2 scored significantly lower than group 3, t = −2.636, df = 307.06, *p* = 0.024, d = 0.289. Furthermore, groups 2.1 and 2.2 were compared on their MD levels. No statistically significant differences were found (t = −0.352, df = 166.28, *p* = 0.725). These results confirm hypothesis H5 (see Table 5).

### 3.8. Number of Aggression Contexts and Their Relationship with MD

To confirm that the differences in MD between the profiles were not exclusively due to the frequency of aggression, we conducted an analysis of covariance (ANCOVA) considering the independent variable as the grouping of the sample by the number of aggression contexts (none, one, and both), and the covariates as the frequency of aggression at school and the frequency of aggression during leisure time.

The analysis revealed a significant effect of context on MD, F(2, 859) = 13.392, *p* < 0.001, η^2^ = 0.028, with a small effect size. The mean MD scores were: 15.11 (SD = 4.75) for the “none” group; 17.44 (SD = 5.97) for the “school or leisure” group; and 19.64 (SD = 6.44) for the “both contexts” group. The covariate of frequency of aggression at school also had a significant effect, F(1, 859) = 8.235, *p* = 0.004, η^2^ = 0.009, but the context of aggression during leisure time did not show significant effects, F(1, 859) = 1.671, *p* = 0.197, η^2^ = 0.002. These results partially confirm hypothesis H6.

In summary, we obtained two unidimensional subscales of 13 items, with adequate reliability values and evidence of validity for their use in similar samples (significant differences between boys and girls, as well as significant correlations between both subscales and MD). In addition, the results allowed us to identify three participant profiles according to the presence of high aggressive behaviors: (1) no high aggression in any context, (2) high aggression in a single context (school or leisure), and (3) high aggression in both contexts. The analyses showed that those who displayed high aggression in both contexts exhibited a significantly higher frequency of aggressive behaviors compared to those limited to only one, both at school and during leisure time. Likewise, aggression patterns clearly differed between the groups. Finally, we observed that participants with high levels of aggression in both contexts obtained significantly higher scores in moral disengagement (MD) than those with high aggression in only one context or none. However, no significant differences in MD were found between those who showed high aggression only in leisure and those who showed it only in the school context. Moreover, the effect of the number of aggression contexts on MD remained significant even when controlling for the frequency of aggressive behaviors, confirming that context exerts its own influence on this mechanism.

## 4. Discussion

The general objectives of this study were to evaluate the psychometric properties of the CEVEO’s aggression subscales in a sample of adolescents from the southern Chile; and analyze the differences in MD between the aggressors’ profiles, taking into account both the frequency of the aggression perpetrated and the number of contexts in which it is carried out.

### 4.1. Factor Structure

The results suggest that the best factor structure is the unidimensional one, which contrasts with the bifactorial organization of the items of the two subscales in the original study by Díaz-Aguado et al. [10]. Although the uni- and bidimensional structures fit adequately and present adequate factor weights and good reliability, the use of the total bullying score offered by the unifactorial model is suggested, both at school and during leisure. In the first case, the factors “exclusion and mean bullying at school” and “severe bullying at school” do not differ sufficiently. The same occurred between the dimensions “mean exclusion and bullying during the leisure” and “severe bullying during leisure”, affecting their discriminant validity. In addition, the unifactorial organization of the items differs from the three-factor structure in Mexico [39], where they differentiated “manifestation of extreme bullying” (F1), “manifestation of severe bullying” (F2), and “manifestation of relational bullying” (F3), considering that in this study, both the school and leisure versions are nested in the same factor.

From a conceptual standpoint, this unidimensional evidence suggests that the items do not reflect qualitatively distinct categories of aggression, but rather different degrees of severity along a single continuum. In this sense, adopting a unidimensional model is consistent with the idea that aggression constitutes a basic construct that may manifest with varying intensity rather than with factorially distinct forms [52]. This interpretation allows for a parsimonious perspective and facilitates the use of the instrument in applied contexts, without losing sensitivity to capture variations in the magnitude of aggressive behavior.

### 4.2. Reliability and Validity Evidence

Both unidimensional subscales presented good internal consistency, in agreement with the reliability results of Díaz-Aguado et al. [10] and Mendoza-González et al. [39]. These findings support the internal consistency of the items, indicating that they homogeneously measure the constructs of school bullying and free-time bullying.

Furthermore, we were able to verify that, although the exercise of PV is greater at school, it also occurs beyond its structure [10,30,31,32,33]. Furthermore, according to Pulido [32] and Pulido et al. [33], the results indicate that those who bully tend to do so in both contexts.

Along with this, and as evidence of discriminant validity according to sex, they indicate that boys report engaging in aggressive behaviors more frequently than girls, both at school and during leisure. These results agree with the evidence reported by international authors [4,7] and in previous studies conducted in Chile [5,6]. In addition, as evidence of validity based on correlation with another construct, the subscales of Bullying at School and Bullying during Leisure presented statistically significant correlations with MD. This result is consistent with several studies that report positive correlations between the two constructs. For example, Gini et al. [16] found a correlation of *r* = 0.25 between MD and aggressive behavior, and Killer et al. [23] reported an *r* = 0.31 between MD and bullying.

### 4.3. Aggressor Profiles

Pulido et al. [33] established peer violence risk profiles using the six CEVEO scales. In this study with a sample of Spanish adolescents, they identified a group with high exposure to violence in both contexts (as victims or aggressors), notably smaller than the rest. This high-risk profile is similar to profile 3 identified in this study, characterized by high levels of aggression in both contexts and representing 18.3% of the sample. This profile not only shows a high frequency of aggressive behaviors, but this frequency is also significantly higher than that observed among participants who exhibit high levels of aggression in only one context (profile 2).

Moreover, these differences are especially marked in certain specific behaviors. In the school context, the percentage difference between profiles is particularly evident in the item “not let participate”, while in the leisure context the item “use threats to scare” stands out. Another relevant finding is the asymmetry between the subprofiles of profile 2: adolescents with high aggression in leisure (profile 2.2) also report notable frequencies of aggression in the school context; in contrast, those with high aggression in school (profile 2.1) show low frequencies in leisure, very close to those of profile 1 (no high aggression).

Taken together, these results suggest that, although those who offend in one context tend to do so in the other [10,32,33], the profiles differ not only quantitatively in terms of aggression frequency, but also qualitatively, underscoring the importance of considering the specific dynamics of each context when analyzing peer violence. In line with this, Díaz-Aguado et al. [10], who, using the same instrument with a sample of Spanish adolescents, identified distinct behavioral patterns depending on the context.

The profile analysis underscores the complexity of the relationship between aggression and MD. Adolescents exhibiting high aggression in both contexts show significantly higher MD scores than those with high aggression in only one context; in turn, the latter present significantly higher scores than those who do not display elevated levels of aggression. Moreover, subprofiles 2.1 and 2.2 do not differ significantly in MD.

Furthermore, the results show that offenders in both contexts score significantly higher on MD, even controlling for the frequency of aggression. These findings suggest two key points. First, elevated MD scores are not solely explained by the frequency of aggressive behaviors but are also influenced by their occurrence across multiple contexts. Second, moral disengagement appears to be cumulative: adolescents who engage in aggression in multiple contexts tend to employ a broader set of cognitive strategies to minimize, justify, or rationalize ethically questionable behaviors.

This finding supports the hypothesis that MD is activated differently depending on the context of aggression [25,26]. As mentioned previously, Bandura’s theory posits that the activation of mechanisms justifying ethically questionable behavior does not occur automatically or uniformly. Factors such as adverse family, school, or community environments [28,29] may facilitate MD activation in one context while leaving other contexts unaffected. Based on this, we interpret that adolescents who engage in violence in both school and leisure contexts, unlike those who engage in high aggression in only one context, have experienced cumulative adversity across multiple life domains, facilitating higher MD scores.

Previous research has shown that exposure to violence at home, at school, in the community, and among peers has both additive and interactive effects on later aggressive behavior [53,54]. Additional studies have identified cognitive and emotional mechanisms linking multi-context victimization to aggression across settings. For example, Dragone et al. [55] highlight the mediating role of self-serving cognitive distortions, while Mrug et al. [56] and Mrug and Windle [54] discuss the impact of desensitization and shifting social norms. Similarly, Ingram et al. [57] and Davis et al. [58], drawing on social learning and problem behavior theories, suggest that repeated exposure to violence contributes to the normalization of aggressive behaviors. Our findings regarding the association between moral disengagement and aggression align with this body of research and underscore the critical role of cognitive processes in legitimizing and sustaining violence.

In short, the results of this study portray the CEVEO subscales as a useful tool to differentiate bully profiles depending on the contextual extent of the bullying: those who bully in both contexts present greater moral disengagement than those who limit bullying to one of them. In short, the approach to the secondary objectives allows us to assert that the bullying subscales of the CEVEO [10] pose an advantage over the OBVQ-R scale successfully adapted to the Chilean context by Gaete et al. [1], although it was not possible to compare the incremental validity of CEVEO directly with the OBVQ-R within the same sample. While the OBVQ-R assesses PV in school and online contexts, the CEVEO bullying subscales allow a more complete approach to the phenomenon by considering bullying in free-time contexts.

### 4.4. Preventive, Clinical and Policy Implications

The results of this study can contribute to the design of school programs aimed at promoting a culture of non-violent coexistence, strengthening socioemotional skills such as empathy, self-control and peaceful conflict resolution, both in school and leisure contexts. By identifying the contexts in which aggression manifests itself, CEVEO can help to adapt promotion strategies to the specific environment, favoring safer and more respectful educational and community climates.

On the other hand, the aggression subscales of the CEVEO are a useful tool for the prevention of peer violence. Its application in educational contexts would allow early identification of students with aggressive behavior, even when it occurs outside the school environment, facilitating interventions before violence becomes chronic. It also makes it possible to focus actions on subgroups at higher risk, such as those who show aggression in both contexts and greater moral disengagement, helping to reduce the likelihood of escalation of the problem.

### 4.5. Limitations

First, we acknowledge that the use of bullying literature to support the aims of this study may raise some conceptual concerns. However, although the CEVEO was originally designed to assess peer violence in school and leisure contexts, the school violence subscale includes several indicators that closely align with the criteria commonly used to define bullying (types of behavior and repetition—explicitly captured through the minimum frequency category “sometimes”). Thus, while the instrument is not presented as an exclusive measure of bullying, it does allow this phenomenon to be considered within a broader conceptual framework of peer violence.

With respect to the study design, three main limitations should be noted: (a) the imbalance in sample composition, with an overrepresentation of municipal (public) schools, which restricts the generalizability of the findings to this type of institution; (b) the reliance on self-report scales, which are susceptible to response biases such as social desirability; and (c) the cross-sectional nature of the study, which prevents examination of the phenomenon’s development over time. In addition, although our results suggest that CEVEO may offer advantages over the OBVQ-R, it was not possible to compare their incremental validity directly within the same sample. Future research should include both instruments in the same dataset to allow a direct assessment of CEVEO’s advantages and its associations with moral disengagement.

Finally, regarding data analyses, three limitations should be considered. First, we decided to drop two items with extremely kurtotic distributions to avoid unreliable parameter estimation. We acknowledge that excluding these very rare behaviors slightly narrows the content domain of the latent construct. Nevertheless, other items with skewed but estimable distributions were retained to preserve construct coverage. Second, the quartile-based strategy used to define profiles does not formally model the full distribution of the data nor allow for the estimation of membership probabilities or measures of uncertainty. This may reduce the precision of interpretation and limit probabilistic inference. Furthermore, it should be noted that the cultural and linguistic adaptation process may have influenced the emergence of a unidimensional structure, and future studies should confirm its stability in other contexts.

### 4.6. Future Research Directions

Subsequent studies should address this phenomenon using representative samples to allow for a broader exploration, including other regions of the country, Indigenous populations, rural areas, and different socio-cultural contexts. From a conceptual perspective, this study could be further strengthened by explicitly operationalizing in the CEVEO the criteria of intentionality to inflict harm and power imbalance, which are commonly employed in the definition of school violence. For example, the definition proposed by Olweus [3] regarding this phenomenon could be incorporated, as is the case in the OBVQ-R [1]. In addition, it is recommended to include both instruments, CEVEO and OBVQ-R, in the same dataset to allow a direct assessment of CEVEO’s advantages and its associations with moral disengagement. Furthermore, bullying should be examined from the perspectives of the two actors not considered in this study, the victim and the bystander, and peer and teacher nomination techniques should be employed to reduce response bias.

## 5. Conclusions

Our findings lead us to conclude that the unifactorial structure is the most suitable for the bullying subscales of the CEVEO in the study sample, in contrast to the original bifactorial structure. This simplifies the process of interpreting the scores. Both subscales showed adequate reliability values, indicating high internal consistency in measuring the constructs. In addition, they present evidence of validity about other variables since a positive and significant association was found between school and leisure bullying scores with moral disengagement scores, a cognitive mechanism that would make it possible to justify violent behaviors, and boys presented higher scores in school and leisure bullying than girls.

Finally, we conclude that the CEVEO subscales are effective in identifying risk profiles based on both the frequency of aggression and the number of contexts in which violence is perpetrated. Of particular concern is Profile 3, which exhibits high-frequency aggression in both contexts. Equally notable is Profile 2, which shows high-frequency aggression in at least one context. These two profiles are especially complex from an intervention standpoint, as they display elevated levels of moral disengagement. Moreover, our findings indicate that these elevated MD scores are associated not only with the frequency of school aggression but also with the number of contexts in which aggression occurs. These results underscore the value of using a tool like the CEVEO to assess aggressive behavior beyond the school setting.

Statement: During the preparation of this work the authors used ChatGPT (version 5.2; OpenAI) in order to improve writing. After using this service, the authors reviewed and edited the content as needed and take full responsibility for the content of the publication.

## Figures and Tables

**Table 1:** Instruments that measure peer violence with psychometric evidence in Chile.

Scale	Sample and Context	Items and Internal Structure	Reliability	Relationship with Other Constructs
Internet Experiences Questionnaire (IEQ) [37]	N = 530 Age = 12 to 19 Context = School and online	28 items with different formats. Factors: 1. Victim of Internet bullying/2. Bullying and/or cyberbullying aggressor/3. Bullying and/or cyberbullying aggressor/4. Victim of bullying/5. Victim of bullying via text message/6. Frequency of Cyberbullying victims	Total IEQ. α = 0.62	Not included
School Peer Bullying Questionnaire (MIAP) [36]	N = 2341 Age = 11 to 17 Context = School	49 Likert format items with 4 response options (frequency). (Sub) Scales: 1. Harassment witness/2. Victim of Bullying/3. Bully and victim of serious bullying/4. Bullying aggressor	Witness: α = 0.86 Victim: α = 0.80 Bully and victim: α = 0.75 Bully: α = 0.72	Not included
Bullying and Victimization Scales [5]	N = 1004 Age = 11 to 13 Context = School	21 Likert format items with 7 response options (frequency). (Sub) Scales: 1. Victimization/2. Bullying	Victimization: α = 0.85 Bullying: α = 0.86	Convergent Validity Victimization and self-perception as a victim: *r* = 0.35 **/Bullying and self-perception as a bully: *r* = 0.46 **.
Bully/victim questionnaire (OBVQ-R) [1]	N = 2775 Age = 9 to 16 Context = School and online	42 Likert format items with 5 response options (frequency). (Sub) Scales: 1. Victimization (10 items)/2. Perpetration (10 items)	Victimization: *α* = 0.91; ω = 0.81 Perpetration: α = 0.92; ω = 0.76	Concurrent validity Victimization and IEQ and MIAP items: *r* = 0.14 *** to 0.36 ***/Perpetration, IEQ, and MIAP items: *r* = 0.22 *** to 0.32 *** (2 items not sig.).

Note. ** *p* < 0.01; *** *p* < 0.001.

**Table 2:** Descriptive analysis of the sample in sociodemographic variables in the total sample and by sex.

	Total Sample (N = 864) n (%)	Boys (n = 363; 42%) n (%)	Girls (n = 501; 58%) n (%)
Type of school based on its funding			
Municipal (Public)	609 (70.5)	234 (64.5)	375 (74.9)
Subsidized Private *	255 (29.5)	129 (35.5)	126 (25.1)
Age			
13 years	70 (8.1)	27 (7.4)	43 (8.6)
14 years	194 (22.5)	79 (21.8)	115 (23)
15 years	222 (25.7)	104 (28.7)	118 (23.6)
16 years	176 (20.4)	69 (19)	107 (21.4)
17 years	147 (17)	61 (16.8)	86 (17.2)
18 years	55 (6.4)	23 (6.3)	32 (6.4)
Region			
Araucanía	452 (52.3)	208 (57.3)	244 (48.7)
Biobío	412 (47.7)	155 (42.7)	257 (51.3)
Income			
Less than 500,000	375 (55.7)	134 (47)	241 (62.1)
500,001–750,000	179 (26.6)	78 (27.4)	101 (26)
750,001–1,500,000	99 (14.7)	57 (20)	42 (10.8)
More than 2,500,001	20 (3)	16 (5.6)	4 (1)
Indigenous People			
Non-Mapuche	587 (67.9)	227 (63.8)	360 (71.9)
Mapuche	262 (30.3)	129 (36.2)	133 (27)

Note. * State-financed and privately managed school.

**Table 3:** Descriptive information of the items and corrected item-total item correlation models 1b and 2b.

Items Bullying at School Subscale		Categories %				M	SD	Skew.	Kurt.	I-T C M1b
		1	2	3	4					
01	Rejecting them	67.2	29.1	3.1	0.6	1.37	0.57	1.47	2.10	0.454
02	Ignoring them	5.9	4.3	6.9	1.9	1.60	0.70	1.06	1.00	0.472
03	Preventing them from participating	88.8	9.1	1.5	0.6	1.14	0.43	3.65	15.29	0.398
04	Insulting them	67.8	23.6	6.4	2.2	1.43	0.71	1.70	2.51	0.597
05	Using nicknames that offend or ridicule them	77.7	16.4	4.3	1.6	1.30	0.62	2.30	5.27	0.595
06	Speaking ill of them	67.7	26.3	4.6	1.4	1.40	0.64	1.68	2.79	0.501
07	Hiding things from them	74.1	21.2	3.5	1.3	1.32	0.60	2.07	4.51	0.429
08	Breaking their things	95	4.3	0.5	0.2	1.06	0.28	5.81	41.24	0.421
09	Stealing things from them	94.9	3.9	0.6	0.6	1.07	0.33	6.02	41.75	0.479
10	Beating them	9.3	8.3	1.2	0.2	1.11	0.37	3.73	16.05	0.502
11	Threatening them to frighten them	94	5.1	0.5	0.5	1.07	0.32	5.51	36.70	0.504
12	Forcing them to do things they do not want to do with threats (bring money, do homework...).	98.4	1.2	0.5	0.0	1.02	0.17	9.16	9.38	0.416
13	Intimidating them with sexual phrases or insults.	95.5	3.4	0.6	0.6	1.06	0.32	6.37	46.20	0.428
14	Forcing them into sexual behavior or situations in which they do not want to participate with threats *.	98.7	0.9	0.2	0.1	1.02	0.16	11.89	163.82	0.236
15	Threatening them with weapons (sticks, knives...) *.	97.3	2	0.2	0.5	1.04	0.26	8.52	82.70	0.230
Items Bullying during Leisure subscale		Categories %				M	SD	Skew.	Kurt.	I-T C M2b
		1	2	3	4					
01	Rejecting them	75.2	21.2	2.4	1.2	1.30	0.57	2.17	5.34	0.523
02	Ignoring them	62	31.1	4.5	2.3	1.47	0.69	1.56	2.47	0.505
03	Not allowing them to participate	88.7	9.3	1.2	0.9	1.14	0.44	3.82	16.88	0.511
04	Insulting them	75.9	17.8	3.9	2.3	1.33	0.66	2.26	5.06	0.633
05	Using nicknames that offend or ridicule them	81.5	15	2.1	1.4	1.23	0.55	2.78	8.62	0.614
06	Speaking ill of them	78.1	18.1	2.4	1.4	1.27	0.57	2.45	6.65	0.556
07	Breaking their things	95	3.8	1	0.1	1.06	0.29	5.38	32.38	0.469
08	Stealing their things	97.2	2.2	0.3	0.2	1.04	0.23	8.15	78.63	0.497
09	Beating them	93.9	5	0.7	0.5	1.08	0.33	5.35	33.77	0.586
10	Threatening them to scare them	93.5	4.9	0.9	0.7	1.09	0.37	5.18	3.49	0.591
11	Forcing them to do things they do not want to do with threats (bring money, do homework...).	98.6	0.9	0.5	0.0	1.02	0.16	9.86	103.84	0.448
12	Forcing them into sexual behavior or situations they do not want to participate in with threats	98.5	1.2	0.1	0.2	1.02	0.19	11.59	155.20	0.342
13	Threatening them with weapons (sticks, knives...)	97.2	1.2	0.5	1.2	1.06	0.36	7.15	52.25	0.485

Note. 1 = Never; 2 = Sometimes; 3 = Often; 4 = Very often; M = Mean; SD = Standard Deviation; Skew. = Skewness; Kurtosis; I-T C = Corrected Item-Total Correlation; M1b = Model 1b; M2b = Model 2b; * = Deleted items.

**Table 4:** AVE, CR, DV indicators of the bi- and unifactorial models of the CEVEO.

CEVEO at School	Model 1a (Bifactorial)		Model 1b (Unifactorial)
	Factor 1: Exclusion and Moderate Bullying at School	Factor 2: Severe Bullying at School	Factor 1: Total Bullying at School
AVE	0.463	0.641	0.514
CR	0.857	0.934	0.932
R^2^_F1-F2_	0.696		-
DV (AVE-R^2^)	−0.214	−0.036	-
CEVEO at leisure	Model 2a (bifactorial)		Model 2b (unifactorial)
	Factor 1: Exclusion and moderate bullying at leisure	Factor 2: Severe bullying at leisure	Factor 1: Total bullying at leisure
AVE	0.602	0.785	0.657
CR	0.900	0.962	0.961
R^2^_F1-F2_	0.677		-
DV (AVE-R^2^)	−0.076	0.108	-

Note. AVE = Average Variance Extracted; CR = Construct Reliability; DV = Discriminant Validity; R^2^_F1-F2_ = squared correlation between F1 and F2.

**Table 5:** Description of aggressor profiles according to the frequency of violence and the context.

		Contexts of Aggression			School Aggression M (SD)	Leisure Aggression M (SD)	MD M (SD)
Profiles		None *n* (%)	One *n* (%)	Both *n* (%)			
1. No high aggression (*n* = 519/60.1%)		178 (34.3)	180 (34.7)	161 (31.0)	13.98 (1.02)	13.51 (0.72)	16.65 (5.76)
2. High aggression in one context (*n* = 187/21.6%)		--	52 (27.8)	135 (72.2)	17.37 (2.95)	15.31 (2.61)	19.48 (5.74)
	2.1 High aggression. School (*n* = 105/56.1%)	--	105 (100)	--	18.98 (2.99)	13.81 (0.81)	19.61 (5.53)
	2.2 High aggression Leisure time (*n* = 82/43.9%)	--	82 (100)	--	15.32 (0.93)	17.23 (2.87)	19.31 (6.04)
3. High aggression in two contexts (*n* = 158/18.3%)		--	--	158 (100)	20.72 (4.48)	20.04 (4.78)	21.30 (6.86)

## Data Availability

Due to ethical and privacy restrictions, the data are not publicly available but may be provided by the corresponding author upon reasonable request.
